# Navigating the combinations of platelet-rich fibrin with biomaterials used in maxillofacial surgery

**DOI:** 10.3389/fbioe.2024.1465019

**Published:** 2024-10-07

**Authors:** Lauma Ieviņa, Arita Dubņika

**Affiliations:** ^1^ Institute of Biomaterials and Bioengineering, Faculty of Natural Science and Technology, Riga Technical University, Riga, Latvia; ^2^ Baltic Biomaterials Centre of Excellence, Headquarters at Riga Technical University, Riga, Latvia

**Keywords:** platelet-rich fibrin, maxillofacial surgery, tissue regeneration, growth factors, bioengineering, 3D printing

## Abstract

Platelet-rich fibrin (PRF) is a protein matrix with growth factors and immune cells extracted from venous blood via centrifugation. Previous studies proved it a beneficial biomaterial for bone and soft tissue regeneration in dental surgeries. Researchers have combined PRF with a wide range of biomaterials for composite preparation as it is biocompatible and easily acquirable. The results of the studies are difficult to compare due to varied research methods and the fact that researchers focus more on the PRF preparation protocol and less on the interaction of PRF with the chosen material. Here, the literature from 2013 to 2024 is reviewed to help surgeons and researchers navigate the field of commonly used biomaterials in maxillofacial surgeries (calcium phosphate bone grafts, polymers, metal nanoparticles, and novel composites) and their combinations with PRF. The aim is to help the readers select a composite that suits their planned research or medical case. Overall, PRF combined with bone graft materials shows potential for enhancing bone regeneration both *in vivo* and *in vitro*. Still, results vary across studies, necessitating standardized protocols and extensive clinical trials. Overviewed methods showed that the biological and mechanical properties of the PRF and material composites can be altered depending on the PRF preparation and incorporation process.

## 1 Introduction

Platelet-rich fibrin (PRF) is an autologous biomaterial derived from venous blood via centrifugation without additives. This protein matrix contains over 1,500 bioactive molecules at up to 600 times the concentration of normal venous blood (Pavlovic et al., 2021; Peck et al., 2015). Since its first reports in 2001, PRF has gained significant interest in regenerative biomaterials for use in oral, maxillofacial, orthopedic, and gynecological surgeries (Dohan et al., 2006; Grecu et al., 2019; Wang et al., 2021). PRF is a second-generation platelet concentrate, succeeding platelet-rich plasma (PRP) (Dohan et al., 2006). Although PRP also has a high concentration of growth factors and potential healing properties, it requires an anticoagulant for preparation (Le et al., 2018). This leads to faster platelet activation and growth factor release, with 95% of growth factors being released shortly after contact with the anticoagulant (Miron et al., 2017).

Different PRF types vary based on preparation protocols, particularly centrifugation speed and time thus resulting in different platelet concentrations. The main types are leukocyte and platelet-rich fibrin (L-PRF), Injectable-PRF (I-PRF), and Advanced-PRF (A-PRF) (Dohan et al., 2006; Miron et al., 2017; Dohan et al., 2014; Ghanaati et al., 2014; Fujioka-Kobayashi et al., 2017). The first PRF preparation method, sometimes called Choukroun PRF or L-PRF (further in text L-PRF), requires 10 mL of a blood sample, that is centrifuged in glass-coated plastic tubes without any anticoagulant for 10 min at 400 × g (g stands for gravitational force) (Dohan et al., 2006). Future modifications to this protocol allowed the development of A-PRF by reducing centrifugation speed and increasing the centrifugation time to 14 min. This method increased the number of immune cells and platelets in the fibrin matrix compared to L-PRF (Ghanaati et al., 2014). A-PRF+ is similar to A-PRF, except the protocol suggests using only 200 × g for 8 min and proves to be even richer in growth factors than A-PRF (Fujioka-Kobayashi et al., 2017). I-PRF is in liquid form compared to other PRF types. The original I-PRF protocol reported in 2015 consists of horizontal centrifugation of 3,300 rpm for 2 min (Almeida Barros Mourão et al., 2015), but 2 years later in a publication by Miron, it was described using 700 rpm (60 × g) for 3 min (Miron et al., 2017). Although different PRF types have progressed over time to improve the biological and mechanical properties of the previous generations, the most used remains L-PRF (Barbosa et al., 2023).

Although PRF is biologically active, it lacks the necessary mechanical properties for soft and hard tissue renewal (Isobe et al., 2017). Studies indicate that Young’s modulus of L-PRF ranges from 187.6 ± 82.73 kPa in membrane form to 30.2 ± 16.7 kPa or lower in clot form (Lara et al., 2023; Haghparast-Kenarsari et al., 2024) In contrast, biomechanical studies on human cadavers show Young’s modulus of oral soft tissue ranges from 8 to 37 MPa, depending on the intraoral tissue site (Choi et al., 2020). To enhance PRF’s mechanical properties, it can be combined with synthetic and natural polymers, calcium phosphates, metals, and various composites. The choice of material to be combined with PRF should depend on the mechanical properties as well as biocompatibility, porosity and other requirements according to the targeted tissue repair.

Calcium phosphate (CaP) materials are widely used for bone tissue substitutes due to their high biocompatibility, osteoconductivity, and resemblance to bone composition (Jeong et al., 2019). Natural polymers offer high bioactivity and low immune response but suffer from poor thermal stability and mechanical strength, making them challenging for shaping and degradation control. Synthetic polymers exhibit suitable mechanical properties for bone tissue regeneration but have poor cell adhesion as they lack appropriate surface-free energy and cannot bond with human tissue (Gao et al., 2017). To overcome these limitations, biomaterials are frequently combined or modified with bioactive substances like growth factors for which PRF can be used (Bjelić and Finšgar, 2021; Fernandez-Medina et al., 2023).

**FIGURE 1:**
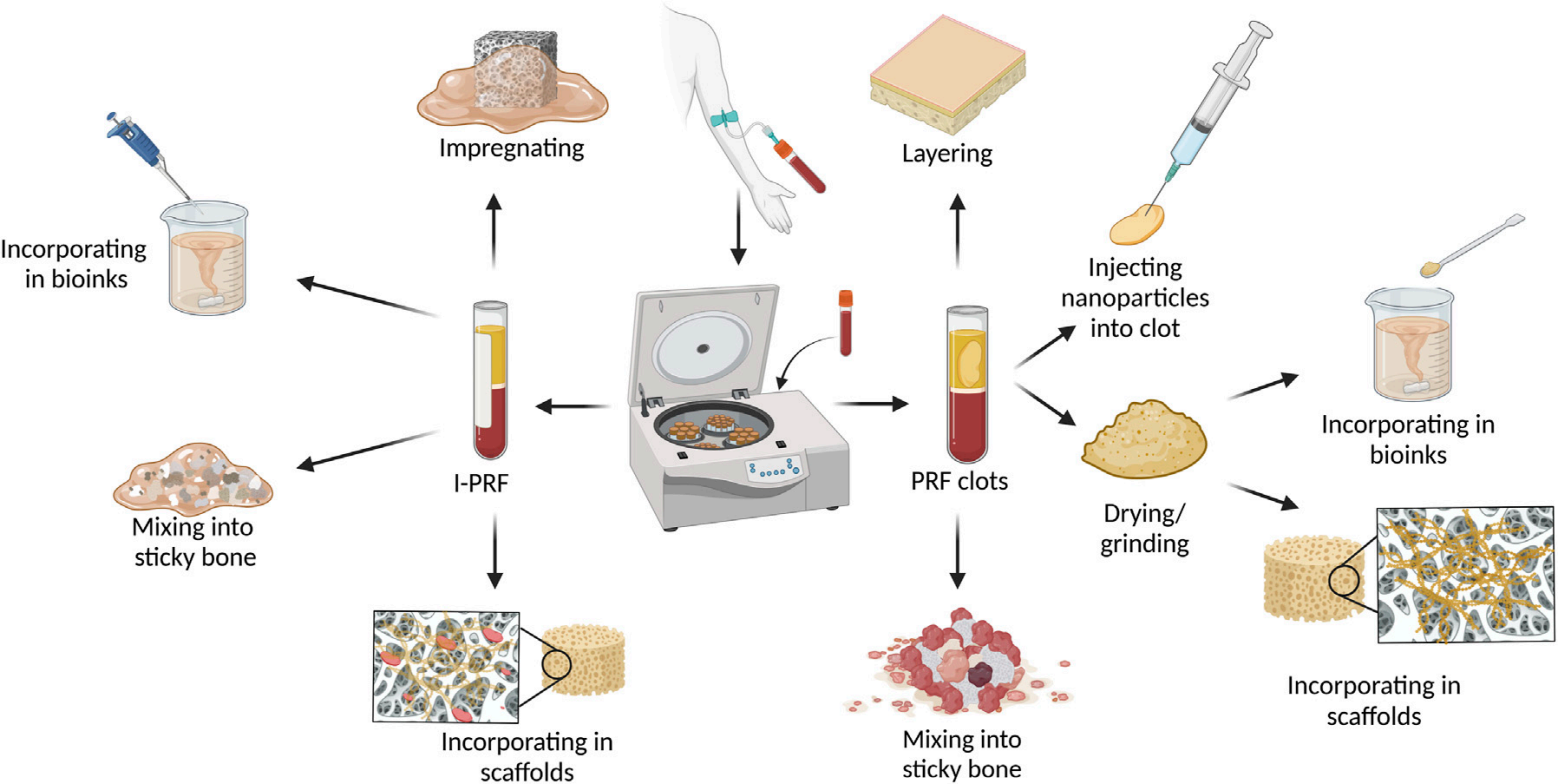
Methods and techniques to prepare PRF and biomaterial composites. Figure created with biorender.com.

Due to PRFs gelatinous structure, it is moldable, allowing users to process it in various ways based on the desired composite outcome as illustrated in Figure 1. The most used PRF types with other biomaterials are those that clot during centrifugation (L-PRF and A-PRF). After pressing PRF into the membrane it can be combined with other membranes, placed in the middle of scaffold layers, or minced and mixed with sponges (Pandikanda et al., 2019; Zhang L. et al., 2019; Sebastian et al., 2022). For incorporation into bioinks or scaffold solutions, lyophilized clot PRF, supernatant from PRF clots, minced PRF clots, and decellularized PRF (dPRF) can be used (Sui et al., 2023; Song et al., 2018; Chi et al., 2019; Tarif et al., 2023) Nanoparticles can be added and incorporated into PRF clots by adding them to the whole blood before centrifugation or by injecting nanoparticle solution into PRF clots (Khorshidi et al., 2018; Ghaznavi et al., 2019; Zalama et al., 2021). PRF clot fibrin structure can also be mineralized by adding alkaline phosphatase (ALP) to whole blood before centrifuging and incubating the PRF membrane for 3 days in calcium glycerophosphate (Douglas et al., 2012; Gassling et al., 2013). A widely used material called “Sticky bone” consists of biomaterial granules mixed with a platelet concentrate to create a moldable biomaterial. Sticky bone is the most common PRF/graft material composite in maxillofacial surgery and can be prepared by adding minced PRF clot, I-PRF, or both, to bone substitute biomaterial granules or particles. (Ramamurthy et al., 2022; Ponte et al., 2021; van Orten et al., 2022; Park et al., 2023; Feng et al., 2022).

Due to the I-PRF liquid nature, it can be used differently than PRF clot types. For scaffold preparation, I-PRF can be added straight to the 3D printing bioinks without any pre-processing like it would be needed for PRF clot types (Yi et al., 2022). Another advantage of I-PRF’s liquid form is its ability to impregnate porous materials like membranes and scaffolds (Patra et al., 2022).

Studies have explored the biological and mechanical properties of biomaterial and PRF combinations, but no review has summarized the effects of these composites to summarize their interactions (Al-Maawi et al., 2021; Blatt et al., 2020; Sampaio et al., 2023; Dambhare et al., 2019). This review aims to combine existing information about PRF usage in association with other biomaterials from *in vitro*, *in vivo*, and clinical studies in maxillofacial and oral surgery. This is done by summarizing articles from academic databased such as PubMed/MEDLINE, ScienceDirect, and Scopus in a time frame from 2013 to 2024, that involves the combinations of PRF with a biomaterial and is tested *in vitro*, *in vivo,* and in clinical studies. To limit the amount of included articles, studies in any way incorporating PRF within the material or *vice versa* are included. Studies using PRF and materials separately in the same defect are not included. This helps readers by summarizing information from open-source databases, providing easier navigation in the field of PRF and biomaterial composites. Thus, this article serves as a guide for selecting suitable composites for planned research or medical cases.

## 2 Inorganic materials

### 2.1 Calcium phosphate ceramics and bone grafts

Bone grafts can be autogenous, allogeneic, xenogeneic, or alloplastic. Autogenous grafts are taken from a patient’s rib or iliac crest allogeneic grafts are harvested from donors, while xenografts come from different species, typically pigs or bovines. Alloplastic grafts are synthetic and made from minerals similar to bone (Kumar et al., 2013). Calcium Phosphates (CaP) are minerals containing Ca_2+_ cations and inorganic phosphate anions, and are the main minerals in bone and tooth enamel, thus they are favored in regenerative surgery (Terzioğlu et al., 2018; Ma et al., 2023; Eliaz and Metoki, 2017). Commonly used CaP in clinical settings include hydroxyapatite (HA), tricalcium phosphate (TCP), and biphasic calcium phosphate (BCP) (Gao et al., 2017). Among the studies, different PRF and CaPs composites are the most frequently studied.

Multiple *in vitro* studies show that combining PRF with allogenic, alloplastic, and xenogenic bone substitute materials (BSM) enhances angiogenic, non-cytotoxic, and osteogenic properties (Blatt et al., 2021a; Blatt et al., 2021b; Kumar et al., 2021; Kumar et al., 2019) Table 1. I-PRF with these bone grafts reduces early platelet-derived growth factor (PDGF) release up to two-fold compared to A-PRF (Blatt et al., 2021a). L-PRF improves cell viability, proliferation, migration, and extracellular matrix formation on alloplastic and xenogenic BG, with higher PRF concentrations yielding better effects (Blatt et al., 2021b). L-PRF combined with dentin chips induces higher dentin sialophosphoprotein expression in primary human dental pulp stem cells (DPSCs) compared to L-PRF with nano-hydroxyapatite (nHA), although nHA + L-PRF induces higher DPSCs mineralization L-PRF with dentin chips (Girija and Kavitha, 2020). Kumar et al. found that mixing cut L-PRF membrane with biphasic calcium phosphate (BCP) inhibits the mitogen-activated protein kinase (MAPK) signaling pathway, reducing osteoclastic effects and osteoclast differentiation (Kumar et al., 2021; Kumar et al., 2019). Combining I-PRF with allogenic, alloplastic, and xenogenic BG results in up to a two-fold increase in new blood vessel formation within 24 h compared to native materials, attributed to elevated PDGF levels. This can be attributed to the authors' findings of elevated Platelet-Derived Growth Factor (PDGF) levels in these instances (Blatt et al., 2021a). Higher PDGF-D concentrations (100 ng/mL) enhance endothelial progenitor cell migration, adhesion, and tube formation (Zhang J. et al., 2019). Additionally, mixing nHA or dentin chips with L-PRF increases the radiopacity of the platelet concentrate, making it more visible in X-ray imaging. (Mahendran et al., 2019).

**TABLE 1:** Summarized results from studies mixing PRF with ceramic materials and bone grafts.

	Materials	PRF preparation protocol	Groups	Type of incorporation	Effect of PRF and biomaterial combinations	Ref.
*In vitro*	BSM granules: Maxgràft^®^, maxresob^®^, Cerabone^®^, BioOss^®^	PRF membrane (10 mL, 1,200 rpm for 8 min) and liquid PRF (protocol not specified)	Materials vs. Materials with PRF Vs. PRF	Mixed	↓ PDGF early release, = TGFβ, VEGF early release Maxgràft®: = Number of blood vessels = Number of blood vessel branching points maxresob®: ↑ Number of blood vessels = Number of blood vessel branching points Cerabone®: ↑ Number of blood vessels ↑ Number of blood vessel branching points BioOss®: = Number of blood vessels = Number of blood vessel branching points	Blatt et al. (2021a)
	BSM granules: Maxgràft^®^, maxresob^®^, Cerabone^®^, BioOss^®^	PRF membrane (10 mL, 1,200 rpm for 8 min) and liquid PRF (protocol not specified)	Materials vs. Materials with PRF	Mixed to obtain sticky bone	Maxgraft®: = HOB viability = HOB proliferation = HOB migration = Osteogenic differentiation = Alkaline phosphatase activity Maxresob®: = HOB viability = HOB proliferation = HOB migration = Osteogenic differentiation = Alkaline phosphatase activity Cerabone®: ↑ HOB viability = HOB proliferation = HOB migration = Osteogenic differentiation = Alkaline phosphatase activity BioOss®: = HOB viability = HOB proliferation = HOB migration = Osteogenic differentiation = Alkaline phosphatase activity	Blatt et al. (2021b)
	BCP particles: 40% β-TCP, 60% HA	Choukroun PRF (protocol not specified)	Material vs. PRF vs. Material + PRF	mixed	↑ Apoptotic pathway activation = TRAP activity ↓ Osteoclastogenesis	Kumar et al. (2019)
	BCP particles: 40% β-TCP, 60% HA	10 mL, 3,000 rpm, 12 min. Clot was pressed to make a membrane and minced	Material vs. PRF vs. Material + PRF	Mixed	↓ TRAP activity ↓ Proinflammatory cytokine release in osteoclast culture ↓ Osteoclastogenesis ↓ MAPK signaling pathways	Kumar et al. (2021)
	BioOss^®^, particles	700x g, 8 min. Glass tubes for solid-PRF, plastic tubes for liquid-PRF	Material + solid-PRF vs. Material + liquid-PRF Vs. Material + solid- and liquid-PRF	Mixed	For material + solid- and liquid-PRF: ↑ Fracture strength ↑ Human osteoblast ↑ Alkaline phosphatase activity ↑ Osteoblast differentiation marker expression ↑ Human osteoblast mineralization = Human osteoblast proliferation ↓ Degradation time *in vitro*	Feng et al. (2022)
	Nano-hydroxyapatite, dentin chips	10 mL, 3,000 rpm, 10 min	Control vs. PRF vs. PRF + nHA vs. PRF + DC	Mixed	PRF + nHA: ↑ HDPCs mineralization PRF + DC: ↑ Proinflammatory cytokine release ↑ HDPCs mineralization	Girija and Kavitha (2020)
	Nano-hydroxyapatite, dentin chips	10 mL, 3,000 rpm, 10 min	Control vs. PRF vs. PRF + nHA vs. PRF + DC	Mixed	↓ Cell viability	Mahendran et al. (2019)
*In vivo*	β-TCP particles	4 mL, 3,000 rpm, 12 min	Control vs. PRF vs. Material + PRF	Mixed	↑ Bone density in early time points ↑ Bone volume in early time points	Abdullah (2016)
	β-TCP particles	10 mL, 400x g, 10 min	Control vs. PRF vs. Material vs. Material + PRF	Mixed	↓ Bone healing time	Yilmaz et al. (2014)
	BCP particles	10 mL, 2,700 rpm, 12 min. Minced clot	Blood vs. BCP vs. BCP + PRF	Mixed	↑ Osteoid percentage in the defect in all time points ↑ Matured bone = Number of osteoblast cells = Number of osteoclast cells	Alkafarani and Baban (2019)
	Nano-hydroxyapatite	1 mL, 700 rpm, 3 min	Control vs. PRF vs. Material vs. Material + PRF	Mixed	↑ Alkaline phosphatase activity ↑ New bone formation = Osteocalcin expression ↓ TRAP activity	Pascawinata et al. (2023)
	Demineralized freeze-dried bone allograft particles	5 mL, 3,000 rpm, 12 min	DFDBA + saline solution vs. DFDBA + rifamycin vs. DFDBA plus PRF	Mixed	↑ Bone-to-implant contact ↑ New bone formation	Şimşek et al. (2016)
	Maxresorb (40% β-TCP, 60% HA) particles	5 mL, 3,000 rpm, 12 min	PRF vs. PRF + material	Mixed	↑ Bone density	Nacopoulos et al. (2014)
	BCP granules (30% β-TCP, 70% HA)	5 mL, 3,000 rpm, 10 min	PRF vs. Material + PRF	Mixed	↑ Bone isoenzyme of alkaline phosphatase in early time points = Nitric oxide in the blood ↓ TRAP activity	Shevchenko and Rublenko (2022)
	Straumann^®^ bone ceramic (40% β-TCP, 60% HA) particles	10 mL, 3,000 rpm, 10 min	Control vs. PRF vs. Material vs. Material + PRF	Mixed	↑ New bone formation	Acar et al. (2015)
	4Bone (BCP: 40% β-TCP, 60% HA)	400x g, 12 min. Minced clot	Control vs. PRF vs. Material vs. Material + PRF	Mixed	↑ New bone formation = Residual amount bone substitute material	Bölükbaşı et al. (2013)
	Powder-type tooth biomaterial	10 mL, 400x g for 12 min	Control vs. PRF vs. Material vs. Material + PRF	Mixed	↑ Implant stability = Regenerated bone area = Bone-to-implant contact (material vs. Material + PRF)	Hwan Jung et al. (2020)
	Bovine HA granules	2 mL, 400x g, 10 min	Control vs. PRF vs. Material vs. Material + PRF	Mixed	= New bone quantity	Knapen et al. (2015)
	Particles: autogenous material, Bio-oss^®^, β-TCP	8 mL, 3,000 rpm, 10 min	Control vs. PRF vs. Autogenous material vs. Autogenous material+ PRF vs. Bio-oss^®^ vs. Bio-oss^®^ + PRF vs. β-TCP vs. β-TCP + PRF	Mixed	= New bone formation = Fibrosis	Karayürek et al. (2019)
	Powder-type tooth biomaterial	10 mL, 400x g for 12 min	Control vs. PRF vs. Material vs. Material + PRF	Mixed	= New bone formation = Bone volume = Percentage bone volume = Bone surface density	Lee et al. (2021)
	β-TCP particles	Not specified	Control vs. Material vs. Material + PRF	Mixed	= Newly formed bone	Kamal et al. (2017)
	Mineralized plasmatic matrix (MPM), BCP alloplast	MPM (10 mL, 2500rpm, 15 min), PRF (10 mL, 3,000 rpm, 10 min)	MPM + BCP vs. BCP + PRF vs. control	Mixed	BCP + PRF: ↑ Collagen amount ↓ Bone surface area ↓ Osteopontin	Anwar and Hamid (2022)
	BioOss^®^ granules	10 mL, 3,000 rpm, 10 min	Material + PRF with colagen membrane vs. material + PRF vs. Material with collagen membrane vs. material	Mixed	Material + PRF with colagen membrane: ↑ Vital mineralizes tissue ↓ Nonmineralized tissue	Maia et al. (2019)
	Deproteinized porcine bone mineral, collagen membrane	10 mL, 1,300 rpm, 8 min. A-PRF tubes for clot type PRF, I-PRF tubes for liquid PRF	DPBM + collagen membrane vs. DPBM + i-PRF Vs. DPBM + i-PRF with PRF membrane	Mixed	= Alveolar ridge dimensions = Residual material = Mineralized tissue = Fibrovascular Tissue = Growth factor gene expression	Park et al. (2023)
Randomized clinical Studies	Hydroxyapatite, alendronate	Not Specified	Control vs. PRF vs. HA +PRF vs. Aledronate + PRF	Not Specified	↑ Change in bone volume	Tiwari et al. (2020)
	BioOss^®^ particles	L-PRF (3,000 rpm, 10 min)	Material vs. Material + PRF	Mixed	↑ New bone formation ↓ Residual graft ↓ Fibrous tissue	Pichotano et al. (2018)
	BioOss^®^ particles	700 rpm, 60 g, 3 min	Material + PRF with collagen membrane vs. Material + PRF	Mixed	↑ Horizontal ridge width in the group without collagen membrane = Vertical bone height = Vestibular depth ↓ Width of keratinized tissue in the group without collagen	Ramamurthy et al. (2022)
	Calcium sulfate, TCP	A-PRF, 1,500 rpm, 14 min	Calcium sulfate + PRF vs. TCP + PRF	Mixed	= Bone gain = Bone reduction	Amam et al. (2023)
	β-TCP particles	10 mL, 3,000 rpm, 10 min	PRF vs. Material vs. Material + PRF	Mixed	↑ Bone density ↑ Bone height = Bone width	hamuda et al. (2023)
	BioOss^®^ particles	L-PRF (400x g, 12 min) Pressed into the membrane and minced	Material vs. Material + PRF	Mixed	= Newly formed bone = Bone-to-graft contact = Fibrous tissue ↓ Residual bone graft	Nizam et al. (2018)
	BioOss^®^ particles	3,000 rpm, 300g, 10 min	Material vs. Material + PRF	Mixed	↑ Newly formed bone = Fibrous tissue = Implant stability ↓ Residual graft	Pichotano et al. (2019)
	Hydroxyapatite, nano-hydroxyapatite	2,500 rpm, 10 min	HA vs. Nano-HA vs. Nano-HA + PRF	Mixed	↑ Reduction in defect size	Elkholly et al. (2022)
	Boneceramic™ particles (60% HA, 40% β-TCP)	Solid-PRF (1,500 rpm, 196x g, 10 min) Liquid-PRF (2,700 rpm, 3 min)	PRF vs. Material vs. Material + PRF	Mixed	↑ Connective tissue ↓ New bone formation ↓ Residual material	Ponte et al. (2021)
	β-TCP particles	Not specified	Control vs. PRF vs. Material vs. Material + PRF	Mixed	↑ Bone density ↓ Bone height reduction = Bone width	Mbarak et al. (2023)
	β-TCP	PRF (2,700 rpm, 12 min), PRP (10 mL, 1. 900 rpm, 5 min; 2. 1,500 rpm, 15 min)	Control vs. Material + PRP vs. Material + PRF	Mixed	↑ Bone with (For material + PRF) ↑ Bone density (For material + PRF) = Bone height	abou shabana et al. (2023)
	Freeze-dried bone allograft	A-PRF (1,300 rpm, 200x g, 8 min)	Control vs. PRF vs. Material vs. Material + PRF	Mixed	= Bone density ↓ Loss of ridge height ↓ Loss of ridge width ↓ Residual graft	Clark et al. (2018)

*In vivo* studies on animal models such as sheep, pigs, rats, rabbits, and dogs have shown that combining PRF with various calcium phosphate (CaP) bone grafts enhances osteoblast activity and accelerates new bone tissue formation, thereby reducing healing time (Abdullah, 2016; Yilmaz et al., 2014; Alkafarani and Baban, 2019; Pascawinata et al., 2023; Şimşek et al., 2016; Nacopoulos et al., 2014; Shevchenko and Rublenko, 2022; Acar et al., 2015; Bölükbaşı et al., 2013). For example, using L-PRF and BCP sticky bone to fill bone defects in sheep resulted in 42% defect coverage by new bone on day 20% and 54.9% on day 40, compared to 29.6% and 49.1% for BCP alone (Bölükbaşı et al., 2013). Similarly, Hwan Jung et al. found that L-PRF mixed with dentin powder improved implant stability and increased regenerated bone area and bone-to-implant contact after 8 weeks (Hwan Jung et al., 2020). Yuan et al. show that PRF in combination with deproteinized bovine bone mineral (DBBM) had higher osteoclast activity than just DBBM in a canine model (Yuan et al., 2021). In rabbits, L-PRF combined with autografts and xenografts promoted faster new bone formation after 8 weeks, though this effect was not observed with β-tricalcium phosphate (β-TCP). (Karayürek et al., 2019). Additionally, some studies show that PRF does not improve healing time when combined with graft materials (Park et al., 2023; Knapen et al., 2015; Lee et al., 2021; Kamal et al., 2017). Knappen et al. observed similar healing patterns in rabbit calvaria between L-PRF, bovine HA, and their combination at early time points of 1, 5, and 12 weeks (Knapen et al., 2015).

Clinical trials testing PRF combined with graft materials for bone defects have been conducted since 2010 (Inchingolo et al., 2010). Multiple studies have since shown that PRF with BSM accelerates dental implant stabilization and tissue healing after sinus lifts, ridge preservation, or bone augmentation have shown positive results in various *in vivo* studies and case reports (Ramamurthy et al., 2022; van Orten et al., 2022; Tiwari et al., 2020; Pichotano et al., 2018; Amam et al., 2023; hamuda et al., 2023; Simone et al., 2018; Massuda et al., 2023; Caramês et al., 2022; Alberto et al., 2020).

Residual bone grafts also tend to degrade quicker when BG is combined with PRF (Nizam et al., 2018). Pichotano showed that after L-PRF + DBBM usage in maxillary sinus augmentation, the residual bone graft material significantly reduced after 4 months in the test group (3.59% ± 4.22%) compared to the control group (13.75% ± 9.99%) with only DBBM in 8 months (Pichotano et al., 2019).

**FIGURE 2:**
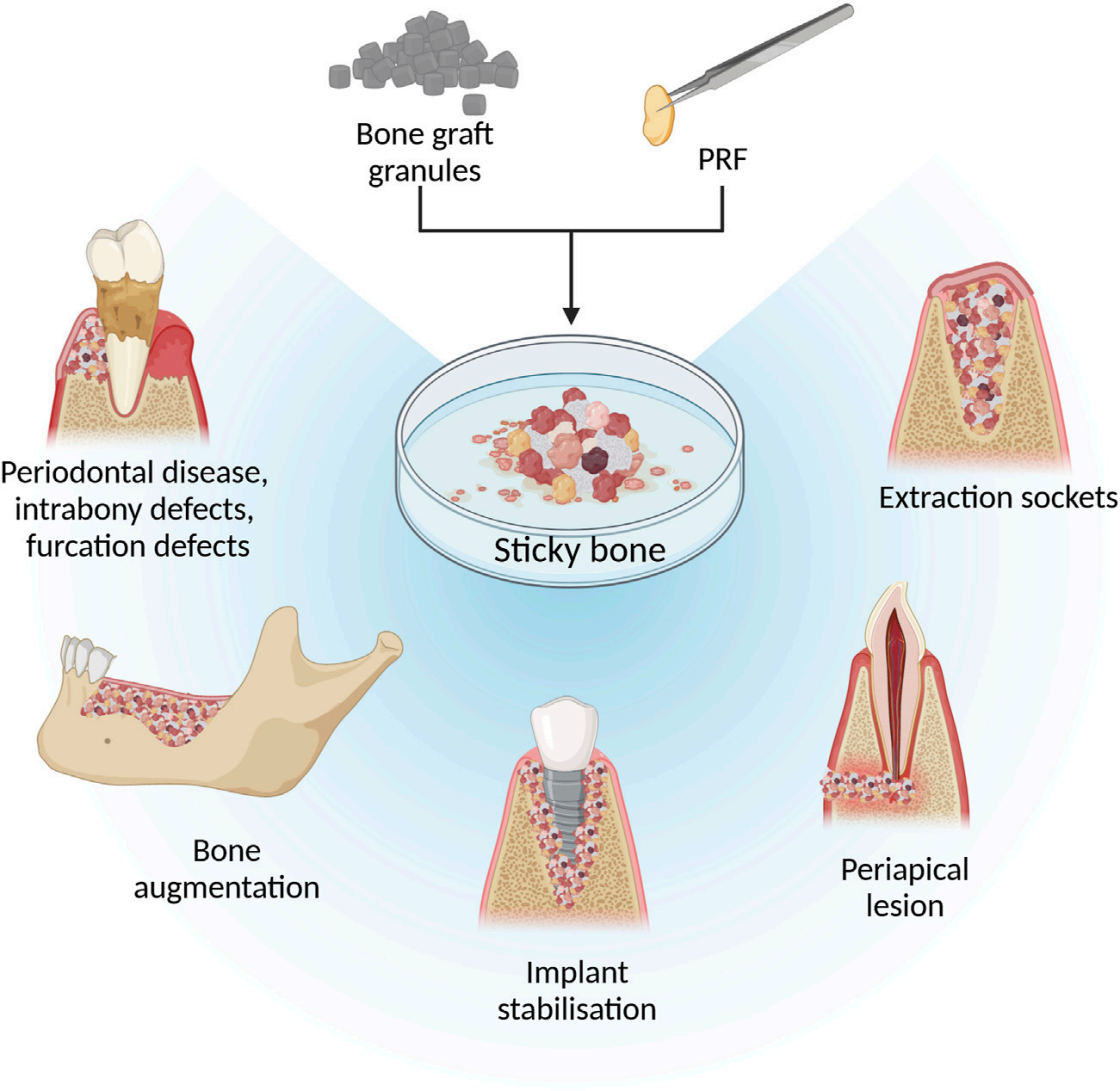
Illustration of using sticky bone in maxillofacial surgeries. Figure created with biorender.com.

In clinical human studies and case reports, sticky bone is one of the most studied PRF and bone graft composites. Sticky bone is widely used for severe bone defects in maxillofacial surgery, which are summarized in Figure 2 (Elkholly et al., 2022; Deenadayalan et al., 2015; Hiremath et al., 2014; Lei et al., 2019; Pradeep et al., 2016; Shivashankar et al., 2013; Lorenz et al., 2018) Feng et al. described that using I-PRF with minced PRF clot for sticky bone preparation shortened solidification time, improved tensile resistance, and prolonged degradation time compared to sticky bone made with each PRF type separately. This preparation method provides a more moldable material for filling difficult bone defects (Feng et al., 2022).

Reports on the efficiency of PRF and biomaterial combinations for extraction socket wound healing are often contradictory. PRF improves healing when combined with β-TCP, but not with BCP. Ponte et al. found that sticky bone made with I-PRF and BCP induced slower new bone formation in 8 months compared to PRF clot or BCP alone, although earlier time points were not tested (Ponte et al., 2021). In contrast, β-TCP mixed with PRF clot improved bone density (620.0 ± 31.02) in 6 months compared to PRF (336.6 ± 66.65) or β-TCP (466.0 ± 38.24) alone and helped sustain alveolar ridge bone height and width (Mbarak et al., 2023). Similarly, β-TCP mixed with L-PRF is more efficient in socket wound healing than β-TCP mixed with platelet-rich plasma in parameters like alveolar bone width, height resorption, and bone density (abou shabana et al., 2023).

In 2019, Clark et al. (2018) noted that a combination of A-PRF and freeze-dried bone allograft (FDBA) for ridge preservation resulted in lower bone mineral density compared to FDBA alone. However, histology showed more vital bone volume with A-PRF and FDBA than with FDBA alone. For horizontal ridge defect treatment, better results are suggested when the sticky bone is made from a mineralized plasmatic matrix rather than L-PRF, showing increased bone surface area, osteopontin expression, and reduced collagen amount by bone maturation (Anwar and Hamid, 2022). Additionally, Maia et al. observed that covering a defect with collagen membranes can reduce the healing efficiency of L-PRF and BSM composites (Maia et al., 2019).

Literature on PRF and bone graft composites for intrabony defects have previously been summarized in multiple specific to this disease-focused meta-analyses and systematic reviews and thus will not be reviewed in this article (Pepelassi and Deligianni, 2022; Ye et al., 2023; Theodosaki et al., 2022). Shortly, 2022 systematic review by Theodosaki et al. (2022) noted that PRF added to inorganic bone grafts (BG) offers small improvements in healing size but faster healing time. Pepelassi and Deligianni (2022)Pepelassi et al. (2022) found that using L-PRF with osseous grafts reduces probing pocket depth and radiographic defect depth while improving clinical attachment levels in endosseous and class II furcation defects for non-smoking chronic periodontitis patients. However, a 2023 meta-analysis by Ye et al. (2023) showed insignificant differences in clinical outcomes between PRF + biomaterials and biomaterials alone. Couple of systematic reviews and meta-analyses focusing on PRF and bone graft adjunctive usage in sinus augmentation noted that this method has inconclusive results. Significant drawbacks in clinical studies include unstandardized PRF preparation protocols and short follow-up periods (Alotaibi et al., 2020; Liu et al., 2019).

### 2.2 Bioactive glass

Another synthetic bone substitute is bioactive glass, which promotes integration with living cells and facilitates the healing process by bonding both to soft and hard tissue by partly converting to hydroxyapatite (Bi et al., 2013; Wilson and Low, 1992). Bioactive glass is composed of minerals like SiO_2_, CaO, Na_2_O, and P_2_O_5_ (Hench et al., 2000). Available in various forms—particulate, powder, mesh, and cones—it can be molded to suit different needs and thus is used for bone reconstruction in maxillofacial surgery (Krishnan and Lakshmi, 2013; Han et al., 2020). However, bioactive glass and PRF combinations have been less studied than CaP material composites.

A few studies have tested PRF clots combined with bioactive glass for intrabony defects, showing positive effects (Agrawal et al., 2017; saravanan et al., 2019) (Table 2). Agrawal’s 2017 study mixed L-PRF with calcium phosphosilicate putty for filling intrabony defects, observing significantly better defect bone fill in the L-PRF + bioglass group compared to the bioglass group alone after 6 months. However, L-PRF + bioactive glass treatment had insignificant changes in pocket depth, clinical attachment level, and gingival recession compared to bioglass (Agrawal et al., 2017). Other studies also indicated that L-PRF + bioglass has similar efficiency to bioglass alone for treating intrabony defects or gingival recession (Vibhor et al., 2021). Vibhor et al. (2021) study found no statistical differences in treatment efficiency between bioglass and bioglass with L-PRF after three and 6 months postoperatively.

**TABLE 2:** Summarized results from studies mixing PRF with bioactive glass.

	Materials	PRF preparation protocol	Groups	Type of incorporation	Effect of PRF and biomaterial combinations	Ref.
Clinical study	NovaBone putty (calcium phosphosilicate particulate)	Choukroun’s PRF (Protocol not specified)	Control vs. Material vs. Material + PRF	Mixed	↑ Defect fill = Alveolar crest level = Gingival index = Pocket Depth = Clinical attachment level = Gingival recession	Agrawal et al. (2017)
	Perioglas (calcium-silicate bioactive glass)	L-PRF (10 mL, 3,000 rpm, 10 min)	Materia vs. Material + PRF	Mixed	↑ Radiological defect fill ↑ Probing pocket depth ↑ Clinical attachment level	saravanan et al. (2019)
	NovaBone putty (calcium phosphosilicate particulate)	L-PRF (10 mL, 3,000 rpm, 10 min)	Materia vs. Material + PRF	Mixed	= Plaque index = Gingival index = Probing pocket depth = Relative attachment level = Radiographic defect depth	Vibhor et al. (2021)

### 2.3 Metals

#### 2.3.1 Zinc

Ionic zinc (Zn) has attracted attention due to its significant role as a micronutrient in physiological and biological systems, their cost-effectiveness, low toxicity, and usability in drug delivery and bioimaging (Su et al., 2019). These nanoparticles can be synthesized via “Green” methods that include extracting them from plants fungus, bacteria and algae (Kalpana et al., 2018). ZnONPs are used for cancer and antibacterial treatment due to the intracellular reactive oxygen species (ROS) generation (Jiang et al., 2018). Zinc also induces bone tissue formation, influences osteoblast proliferation, collagen synthesis, and ALP activity (Molenda and Kolmas, 2011). In maxillofacial surgery, ZnONPs are added to scaffolds to reduce bacterial biofilm production and enhance implant osteointegration (Pushpalatha et al., 2022).

**FIGURE 3:**
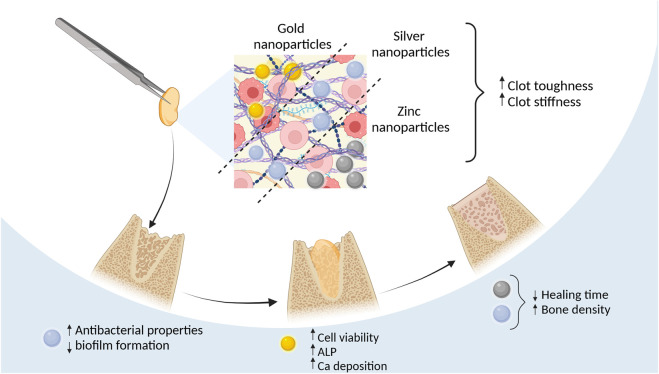
Effects of using metal nanoparticles incorporated into PRF Figure created with biorender.com.

Zalama et al. (2021); Zalama et al. (2022) studied the bone tissue regenerative effects of ZnONPs in size <100 nm by injecting them into L-PRF clots with insulin syringes. In two studies they treated New Zealand white rabbit critical ulnar defects with L-PRF/ZnONPs composite. In their 2021 study, radiographic examinations revealed similar healing scores and new tissue formation for both L-PRF and L-PRF/ZnONPs after 1 and 2 months (Zalama et al., 2021) However, their 2022 study provided a more detailed analysis, demonstrating that L-PRF/ZnONPs outperformed L-PRF alone at all postoperative time points (30, 60, and 90 days) in callus bridging scores, defect size reduction, and bone marrow canal formation. Specifically, L-PRF/ZnONPs promoted higher new bone tissue density at day 60 (1,498.95 ± 77.19 Hounsfield units), comparable to normal bone density (1,508.20 ± 144.52), whereas L-PRF and control groups failed to reach the required bone density even by day 90 (1,212.52 ± 79.18 and 1,284.53 ± 188.30, respectively) (Zalama et al., 2022). Although these studies did not compare L-PRF/ZnONPs to ZnONPs alone, they provided critical insight that combining PRF with ZnONPs significantly improves bone regeneration time and quality compared to PRF alone (Figure 3) (Table 3).

**TABLE 3:** Summarized results from studies mixing PRF with Zink.

	Materials	PRF preparation protocol (min)	Groups	Type of incorporation	The effect of adding PRF to biomaterial	Ref.
*In vivo*	Zinc oxide nanoparticles	3 mL, 3,000 rpm, 10	PRF vs. PRF + ZnONPs vs. control	Zinc oxide nanoparticles injected into PRF clot	↑ Healing score in early time points = Brinding callus score (PRF vs. PRF + ZnONPs)	Zalama et al. (2021)
	Zinc oxide nanoparticles	4 mL, 3,000 rpm, 402× g, 10	PRF vs. PRF + ZnONPs vs. control	Zinc oxide nanoparticles injected into PRF clot	↑ Recreation of the marrow cavity ↑ New bone density ↑ Defect bridging by bicortical callus ↑Bone remodeling score ↓ Defect size	Zalama et al. (2022)

#### 2.3.2 Silver and gold nanoparticles

Silver nanoparticles (AgNP) are known for their antimicrobial, antifungal, and antioxidant properties (Burdus et al., 2018). Their antimicrobial effects are due to targeting cell membranes, generating reactive oxygen species (ROS), and inducing oxidative stress through silver ion release and particle size/form (Yan et al., 2018). However, these effects also occur in human cells, where AgNPs are seen as “non-self” by the immune system, triggering immune responses in a dose-dependent way (Dakal et al., 2016; Pauksch et al., 2014). Despite these challenges, AgNPs are beneficial for bone regeneration, as they promote early bone callus formation by attracting and promoting the proliferation of mesenchymal stem cells (Zhang et al., 2015).

Because of the antimicrobial activity of silver researchers have been interested in the AgNP and PRF composites (Table 4). The addition of AgNPs to LPRF membranes improved tensile strength 2-fold and stiffness 5-fold while the toughness of the L-PRF did not change (Khorshidi et al., 2018). L-PRF modified with AgNP in size <100 nm exhibits superior antimicrobial activity against *Streptococcus*, *Klebsiella pneumoniae,* and *Candida* species and inhibits biofilm formation (Khorshidi et al., 2018; Haddadi et al., 2018). Salih et al. (2018) demonstrated that the L-PRF/AgNPs combination significantly improved bone tissue regeneration speed and quality within 4 weeks compared to each material used separately. Notably, none of these studies reported the cytotoxic effects typically associated with AgNPs.

**TABLE 4:** Summarized results from studies mixing PRF with silver or gold.

	Materials	PRF preparation protocol	Groups	Type of incorporation	Effect of PRF and biomaterial combinations	Ref.
*In vitro*	Silver nanoparticles	L-PRF (10mL, 2,700 rpm, 12 min)	PRF vs. PRF + silver nanoparticles	Nanoparticles added to the blood before centrifugation	↑ Antimicrobial activity ↑ Tensile strength ↑ Stiffness ↑ Toughness	Khorshidi et al. (2018)
	Silver nanoparticles	L-PRF (10mL, 2,700 rpm, 12 min)	PRF vs. PRF + silver nanoparticles	Nanoparticles added to the blood before centrifugation	↓ Biofilm formation with higher silver nanoparticle concentration	Haddadi et al. (2018)
	Gold nanoparticles	A-PRF + (300 rpm, 8 min)	PRF vs. PRF + AuNP	Nanoparticles added to the blood before centrifugation	↑ hMSCs cytotoxicity with higher AuNP concentration ↑ Alkaline phosphatase activity	Ghaznavi et al. (2019)
*In vivo*	Silver nanoparticles	3 mL, 3,000 rpm, 10 min. A clot was pressed to make a membrane	Control vs. AgNP vs. PRF membrane vs. AgNP + PRF membrane	Not mentioned	↓ Bone healing time	Salih et al. (2018)

Similarly, gold nanoparticles (AuNPs) also have osteogenic and bactericidal effects, that are influenced by surface charge, reaction nature, and aggregation level (Basova et al., 2021; Zhang et al., 2021). The exact mechanism behind AuNPs’ antibacterial action remains unclear, with some attributing the effects to co-existing chemicals (Basova et al., 2021). The ability of AuNPs to induce osteogenic differentiation could be promoted by the ability to upregulate bone-related protein (Runx2, Col-1B, OPN, and ALP) expression and cell mineralization (Zhang et al., 2021). Concerns about AuNPs toxicity and long-term safety necessitate further *in vivo* studies to determine biodistribution and potential toxicity (Basova et al., 2021).

Indirect *in vitro* testing using conditioned medium from A-PRF+ (1,300 rpm, 8 min) enriched with 53 ± 2 nm AuNPs increased human mesenchymal stem cell (hMSCs) viability compared to only AuNPs that decreased viability in dose dose-dependent manner with the highest viability being at 0.005 mM and the lowest at 0.5 mM particle concentration. Osteogenic differentiation markers like the ALP in the supernatant from AuNPs/A-PRF+ were significantly higher than in the control and A-PRF+ groups. Alizarin Red staining revealed calcium deposition in human mesenchymal stem cells treated with AuNPs/A-PRF+ conditioned medium, indicating enhanced osteoconduction (Ghaznavi et al., 2019).

## 3 Polymers

### 3.1 Polycaprolactone

Polycaprolactone (PCL) is a semicrystalline, biodegradable polymer derived from petrochemical products (Bezwada et al., 1995). PCL is used for drug delivery and for tissue engineering (e.g., bone, blood vessel, cartilage) due to its non-toxicity, biocompatibility, and long degradation time of 2 to 3 years but it lacks hydrophilic functional groups thus inhibiting cellular growth (Engelberg and Kohn, 1991; Malikmammadov et al., 2018; Fu et al., 2012). Therefore, to enhance biocompatibility, PCL materials are often coated with growth factors or synthetic peptides (Zhang et al., 2009; Qin et al., 2022). In maxillofacial surgeries PCL is used for bone reconstructions in maxilla and mandibulae (Hwang et al., 2023; Naik et al., 2020).

Growth factors from PRF can enhance the biocompatibility of PCL biomaterials (Fernandez-Medina et al., 2023; Al-Maawi et al., 2021). Although hydrophobic, PCL scaffolds can physically bind growth factors on their surface, but the protein absorption quantity depends on material surface roughness and hydrophilicity which depends on the manufacturing process (Khampieng et al., 2018; Shen and Hu, 2021). Fernandez-Medina et al. demonstrated that leukocyte platelet-rich plasma (L-PRP) and I-PRF can induce different protein bindings on PCL surfaces. When immersed in I-PRF, PCL surfaces initially bind high molecular weight (>90 kDa) proteins, which gradually desorb and are replaced by middle-low (50–30 kDa) and low (<30 kDa) molecular weight proteins, such as IL-8, eotaxin, IP-10, and RANTES. Conversely, PCL surfaces coated with L-PRP show stable binding of middle-low and low molecular weight proteins like the γ- and β-chains of fibrinogen, which can induce pro-inflammatory processes (Fernandez-Medina et al., 2023; Luyendyk et al., 2019). PCL scaffolds coated with I-PRF have superior protein corona formation compared to those coated with pure platelet-rich plasma, L-PRP, or plasma, indicating better bioactivity. Notably, the presence of a CaP coating on the PCL surface did not affect the protein corona formation when I-PRF was applied (Fernandez-Medina et al., 2023). The method of PRF production plays a crucial role in the effectiveness of PCL-PRF composites. Al-Maawi et al. (2021) found that PCL meshes coated with I-PRF prepared using a low centrifugation method (44 × g) resulted in higher human primary osteoblast adherence after 7 days compared to a high-speed centrifugation method (710 × g). The low-speed PRF composite released twice as many growth factors over 3 and 7-day periods and promoted higher ALP expression from primary osteoblasts seeded on the PCL scaffold (Table 5).

**TABLE 5:** Summarized results from studies adding PRF to polycaprolactone.

	Materials	PRF preparation protocol	Groups	Type of incorporation	The effect of adding PRF to biomaterial	Ref.
*Ex vivo*	PCL scaffolds, calcium phosphate coating	I-PRF (10mL, 700rpm (60x g, 3min))	I-PRF/PCL/CaP Vs. PLASMA/PCL/CaP vs. I-PRF/PCL	Impregnated	↑ Total protein content on the surface ↑ Adsorption of low-to-medium molecular weight proteins from i-PRF	Fernandez-Medina et al. (2023)
*In Vitro*	OsteoporeTM (PCL mesh)	10 mL, High-RCF protocol (710× g), low-RCF protocol (44× g)	PCL + PRF high-RCF Vs. PCL + low-RCF Vs. PCL	Impregnated	↑ Growth factor release from PCL + PRF low-RCF ↑ Growth factor release in pOB cell culture, from PCL + PRF low-RCF ↑ pOB attachment on scaffolds (↑↑ with PCL + PRF low-RCF) ↑Alkaline phosphatase activity with PCL + PRF low-RCF	Al-Maawi et al. (2021)
*In Vivo*	PCL 3D printed scaffolds	10 mL, 3,000 rpm, 1,670x g, 10 min	PCL vs. PCL + PRF vs. PRF vs. Control (empty defect)	Scaffold added to the blood before centrifugation	= Mineralization volume in the defect (PCL vs. PCL + PRF) = Area of connective tissue	Chen et al. (2021)

The limited number of extensive *in vivo* studies on PCL and PRF composites limits the understanding of their overall effect. One study showed that even after the addition of L-PRF the surrounding bone tissue of rat calvaria did not adhere to these scaffolds and used connective tissue to attach to it. The same report stated that the addition of PRF on PCL has the same effect on new bone formation as native PCL. Their PRF preparation method involved a high-speed centrifugation method (1,670 × g), which could explain the reduced biocompatibility (Chen et al., 2021). Verma et al. (2014) showed that PCL with PRF promotes early bone healing in peri-implant defects. However, this study lacked a control group with native PCL or PCL with a different coating, making accurate interpretation of results difficult.

### 3.2 Collagen and gelatin

Collagen is the most abundant protein in the human body, providing a scaffold for cells and aiding in the transfer of internal and external forces (Meyer, 2019). There are over 27 types of collagens in vertebrates, with type I collagen being the most common in skin, bone, and tendon (Birk and Bruckner, 2005). Gelatin, a collagen derivative, is a biodegradable and biocompatible protein produced by hydrolyzing collagen’s triple helical structure into random coiled domains, resulting in a molecular structure similar to collagen (Shoulders and Raines, 2009; Davidenko et al., 2016). In maxillofacial surgery, collagen is used as a membrane for creating a barrier between soft and bone tissue, scaffolds for dental pulp regeneration and gelatin sponges are used as a space filler and a hemostatic absorbent (Shabat and Yousif, 2021; Debel et al., 2021; Sheikh et al., 2017).

Commercially available collagen membranes interact differently with I-PRF – for example, membranes with smaller pore sizes restrict the flow of PRF through its layers. This results in lower PRF absorption and shallower cell penetration in the material (Al-Maawi et al., 2019). However, collagen-based matrices with loosely arranged fibrils create a porous structure that allows PRF and its cells to be more easily absorbed (Udeabor et al., 2020). It is worth noting that no studies have described how this PRF absorbability affects cytokine release.

Studies show that when commercially available non-cross-linked equine-derived collagen hemostatic sponge is soaked with I-PRF it prolongs the cytokine release by 6 days, but induces proinflammatory cytokine release from PRF (Herrera-Vizcaíno et al., 2020). Compressing A-PRF+ with a collagen membrane, however, results in the highest growth factor release within 24 h (Blatt et al., 2020). Two studies by Blatt et al. (2020) have shown varying results with PRF and collagen combinations (Sebastian et al., 2022). In 2020, they found that pressed PRF (177 × g for 8 min) combined with three different porcine collagen membranes led to more new blood vessels and branching points than native materials (Blatt et al., 2020). In 2022, they reported that porcine- and bovine-derived membranes combined with PRF (177 × g for 8 min) had the same impact on new blood vessel formation as native membranes after 72 h (Sebastian et al., 2022). Hoda et al. (2021) showed that a three-collagen membrane incubated with A-PRF for 2 h increased gingival fibroblast adherence threefold and human osteosarcoma adhesion twofold. This effect was not seen with cell-free human dermal matrix or porcine-origin collagen matrix. A separate study by Park et al. (2018) showed that the addition of L-PRF to a porcine-derived collagen matrix significantly improves cell activity and mature endothelial cell migration by almost 8-fold.

*In vivo* studies and case reports show that I-PRF-soaked collagen matrices provide better results for gingival recession, extraction sockets, and sinus floor augmentation (Santamaria et al., 2023; Michels et al., 2023; Gülşen and Dereci, 2019) (Table 6). For gingival recession treatment, collagen membranes soaked with I-PRF achieved higher overall root coverage by 9.3% after 1 month, 15.6% after 3 months, and 13% after 6 months compared to native membranes (Patra et al., 2022). In another instance, Pandikanda et al. successfully treated oro-antral communication using minced L-PRF mixed with a collagen sponge, resulting in no complications and sustained vestibular depth (Pandikanda et al., 2019).

**TABLE 6:** Summarized results from studies mixing PRF with collagen and gelatin materials.

	Materials	PRF preparation protocol	Groups	Type of incorporation	The effect of adding PRF to biomaterial	Ref.
*Ex vivo*	Mucograft^®^, Bio-Gide^®^, Mucoderm^®^, Collprotect^®^, BEGO^®^	Liquid-PRF (10 mL, 600 rpm, 44 × g for 8 min)	Mucograft^®^ + PRF,vs. Bio-Gide^®^ + PRF vs. vs. Collprotect^®^+ PRF vs. BEGO^®^ + PRF	Impregnated	*No PRF penetration into the membrane:* - BEGO^®^ + PRF *Partial penetration into the membrane*: Bio-Gide^®^ + PRF; Mucoderm^®^ + PRF; Collprotect^®^+ PRF. *Total penetration:* Mucograft^®^ + PRF	Al-Maawi et al. (2019)
	Mucomaix^®^ matrix (collagen and elastin)	I-PRF (10 mL, 700 rpm, 60x g, 3 min)	Material + PRF	Impregnated	Loose collagen fibrils lead to PRF penetration through the material matrix	Udeabor et al. (2020)
*In vitro*	Parasorb fleece HD^®^ (equine-derived collagen matrix)	Liquid-PRF (10 mL, 600 rpm, 44×g, 8 min)	Collagen matrix + PRF vs. Compressed collagen matrix + PRF	impregnated	*Mechanical properties:* ↓ Liquid absorption capacity of the matrix when compressed; *In vitro* *:* ↑ Prolonged cytokine release; ↑ Increase in proinflammatory cytokine release;	Herrera-Vizcaíno et al. (2020)
	Mucoderm^®^ (collagen matrix), Jason^®^ (porcine pericardium), Collprotect^®^ (collagen matrix)	PRF membrane (10 mL, 1,200 rpm, 177x g, 8 min), pressed with “PRF Box”, cut and pressed with “PRF Box”	Materials with PRF Vs. Materials without PRF	Membranes pressed together	↑ Angiogenesis = Growth factor release then just PRF	Blatt et al. (2020)
	Collagen matrixes – Bio-Gide^®^ and Symbios^®^	PRF membrane (1,200 rpm for 8 min, 177× g), pressed with “PRF Box ”	Materials with PRF Vs. Materials without PRF Vs. PRF	Membranes pressed together	Bio-Gide^®^ and Symbios: = Vessels per mm^2^ = Vessel branching points per mm^2^	Sebastian et al. (2022)
	Mucoderm^®^ (collagen matrix), alloderm (cell-free human dermal matrix), three collagen membrane	A-PRF (2 mL, 1,500 rpm, 14 min)	Membranes with A-PRF Vs. membranes without A-PRF	Impregnated	Mucoderm®: = Osteoblast adhesion = Fibroblast adhesion Alloderm: ↓ Osteoblast adhesion = Fibroblast adhesion Three collagen: ↑ Osteoblast adhesion ↑Fibroblast adhesion	Hoda et al. (2021)
	Mucoderm^®^ (collagen matrix)	L-PRF (10 mL, 400× g, 12 min). The clot was incubated with a serum-free medium at 37°C. After 24 h, the medium with PRF exudate was collected.	Enamel matrix derivative Vs. Mucoderm with PRF Vs. Mucoderm	Impregnated with the PRF-conditioned media.	↑ HUVEC proliferation when compared to native membrane ↑ HUVEC Migration = HUVEC attachment between Enamel matrix derivative And PRF	Park et al. (2018)
*In vivo*	Gelatin gel	2.5mL, 3,000 rpm, 10 min. PRF was cut and stirred using a magnetic stirrer (37C, 700 rpm, 24 h. After the gel was precipitated by centri-fugation (4C, 12,500 rpm, 10 min)	Gelatin gel vs. Gelatin gel with PRF	incorporated	↓ Skin defect recovery time = Granulation tissue thickness	Suzuki et al. (2013)
	Gelatin nanoparticles	I-PRF (7 mL 700 rpm for 3 min)	Gelatine nanoparticles Vs. Gelatine nanoparticles with PRF vs. PRF	Mixed with repetitive extrusion	*Mechanical properties:* ↑ Gel strength ↑ Self-healing properties before solidification ↑ Compressive modulus *In vivo* *:* ↑ Bone volume in sinus augmentation model ↑ Number of trabecular bone ↑ Mature laminar bone ↑ prolonged growth factor release ↑ Number, density, and diameter of blood vessels ↓ Gel solidification time ↓ Bone resorption	Mu et al. (2020)
	Gelatin nanoparticles	I-PRF (700 rpm, 3 min)	1.Control group (no grafting material) 2. DBBM group. 3. DBBM + i-PRF group. 4. GNPs group. 5. GNPs + i-PRF group.	Mixed with repetitive extrusion	*Mechanical properties:* ↑ Yield stress when lower PRF concentration *In vitro*: ↓ Whole blood clotting time compared to DBBM, but = to GNPs *In vivo* *:* ↑ Bone density ↑Early osteogenesis ↑Early angiogenesis ↑ Higher osteoclast activity than GNPs, but lower activity than DBBM	Yuan et al. (2021)
Clinical studies	HEALIGUIDE Bio-resorbable membrane (collagen membrane)	I-PRF (10 mL, 700 rpm, 3 min)	Membrane with PRF vs. Membrane with saline	Impregnated	= Plaque index = Gingival index = Probing pocket depth ↓ Recession depth ↓ Recession width	Patra et al. (2022)

Compared to collagen membranes, there are fewer reports on gelatin in combination with PRF. Gelatin gels can effectively carry growth factors from PRF and prolong the growth factor release in the surrounding environment. (Suzuki et al., 2013; Mu et al., 2020). Gelatin nanoparticles (GNPs) are inherently fragile, with a compressive modulus of 9.2 ± 2.7 kPa and a tensile modulus of 14.1 ± 3.1 kPa. However, adding I-PRF to the hydrogel enhances its resistance to compressive (32.7 ± 4.6 kPa) and tensile forces (Elastic modulus 25.3 ± 4.1 kPa). These composite hydrogels exhibited self-healing and shear-thinning properties, making them suitable for injections before complete solidification. In a rabbit model for sinus augmentation, the GNP I-PRF composite led to significantly more new bone formation, better lamellar bone maturation, and improved new bone height and area compared to GNP gel alone over 8 weeks (Mu et al., 2020). Similarly, Yuan et al. (2021) observed that GNPs + I-PRF could be smoothly injected and maintained their form in water for 72 h even after shaking. The 20 w/v% GNPs in I-PRF hydrogel showed the highest toughness, with a yield stress of 33.2 kPa compared to 15 w/v% and 12 w/v% gels. This combination also resulted in higher bone density and blood vessel percentage area of the alveolar ridge in a canine model compared to DBBM and DBBM with PRF. Additionally, positive results were obtained when gelatin sponge pieces were combined with L-PRF for furcation defect treatment, showing improvements in horizontal and vertical clinical attachment and probing pocket depth. However, the study did not include a control group of only gelatin sponges without PRF, making it difficult to assess the specific impact of PRF (Ahuja et al., 2022).

### 3.3 Silk

In nature, silk is produced by certain lepidopteran larvae like silkworms, spiders, scorpions, and flies. Silk fibroin is made of repetitive protein sequences and composed of β-sheet structures (Bini et al., 2004). Silk biomaterials can be improved via amino acid side chain modifications or combining it with different biomaterials, thus allowing for broader applications. These types of materials are biocompatible and useful in wound healing and tissue engineering of bone, cartilage, tendon, and ligament (Vepari and Kaplan, 2007). Although silk is mostly used for sutures, some research groups are using silk proteins to produce scaffolds, hydrogels, and films for tissue repair (Holland et al., 2019).

Since 2013 only one study has investigated PRF combinations with silk biomaterials. This study done on patients showed similar results for implant stability quotients at 3 months between silk fibroin powder + cut PRF clot (type not specified) and PRF alone (57.0 ± 5.29 vs. 58.6 ± 4.95, respectively), but after 6 months the implant stability quotients for silk fibroin + PRF group (76.8 +/−3.65) was significantly higher than PRF (66.80 +/−5.79). Authors observed that bone density after 6 months was significantly higher with Silk fibroin + PRF (418.8 ± 181.3) compared to PRF (345.5 ± 179.5) (Ramy Salah et al., 2021). Although studies show that Silk fibroin with PRF has the potential to help in new bone regeneration studies comparing this composite to native silk fibroin are needed.

## 4 Composites

Composite materials retain the mechanical and biological properties of their components, making them widely used in tissue engineering. They are particularly useful in maxillofacial surgery due to their ability to be shaped for complex bone structures (Huang et al., 2024). An example is the use of 3D printed PCL/β-TCP composite scaffolds for facial bone reconstruction, combining PCL’s mechanical properties with β-TCP’s bone-mimicking characteristics (Jeong et al., 2022). This section focuses on studies where PRF is added to composite materials, analyzing three groups: composites with ceramic materials, polymer-polymer composites, and 3D-printed composite materials.

### 4.1 PRF with inorganic composite materials

Composite materials with inorganic compounds typically consist of polymers like collagen, gelatin, PLGA, and PCL, combined with an inorganic phase such as CaP or metals. This review summarizes 13 studies (Table 7), focusing on the combination of PRF with these materials. The most common method is mixing PRF with CaP to create sticky bone (Peker et al., 2016; Zhang Yue et al., 2023; Bastami et al., 2022), followed by coating synthesized scaffolds via impregnation (Zheng et al., 2015; Espitia-Quiroz et al., 2022) electrically binding to microspheres, or layering in multilayer scaffolds (Zhang L. et al., 2019; Alhasyimi et al., 2017; Alhasyimi et al., 2018; Zhang L. et al., 2023). The impact of PRF on the mechanical properties of these biomaterials is rarely documented. Tarif et al. reported that decellularized PRF coating on strontium-doped porous magnesium phosphate scaffolds did not affect the ultimate compressive strength (Tarif et al., 2023). Beiranvand et al. observed that I-PRF and hydroxyapatite (HA) coating enhanced the hydrophilicity of 3D-printed PCL scaffolds. They also found that platelet concentrate improved preosteoblast viability, doubling cell proliferation after 7 days, and increased RUNX2 gene expression on PCL/HA/PRF scaffolds after 14 days compared to PCL/HA scaffolds (Beiranvand et al., 2022). Similar improvements in cell viability were noted on nHA/PLGA scaffolds impregnated with PRF growth factors, L-PRF-coated HA scaffolds combined with collagen and PLGA copolymer, and L-PRF incorporated into a triple-layer scaffold consisting of an electrospun PCL/gelatin top layer and chitosan/poly (y-glutamic acid)/nHA hydrogel bottom layer (Zhang L. et al., 2019; Zhang L. et al., 2023).

**TABLE 7:** Summarized results from studies incorporating PRF into composites with ceramic materials and composites with polymer materials.

Materials		PRF preparation protocol	Groups	Type of incorporation	The effect of adding PRF to biomaterial	Ref.
Studies incorporating PRF into composites with ceramic materials						
*In vitro*	Scaffold - PCL, HA	I-PRF (700rpm, 3 min)	Control vs. PCL vs. PCL-HA vs. PCL-HA + PRF	Impregnated	Mechanical properties: ↑ Hydrophilicity *In vitro*: ↑ MC3T3-E1 cell viability ↑ MC3T3-E1 cell proliferation ↑ Osteocyte differentiation = Osteogenic differentiation (PCL/HA = PCL/HA/PRF)	Beiranvand et al. (2022)
	Scaffold - NaHA, poly-(D, L-lactic acid-co-glycolic acid), lyophilized PRF	Lyophilized PRF (400xg, 10 min). PRF clot was frozen at −80°C for 30min before being freeze-dried overnight at −51°C.	HA/PLGA vs. HA/PLGA/Gel vs. HA/PLGA + PRF	Impregnated	*In vitro*: ↑ MG63 cell viability ↑ MG63 cell adhesion	Zheng et al. (2015)
	Tetracalcium phosphate (TTCP), gelatin, O-Phospho-l-Serine (OPLS), Lyophilized PRF	Lyophilized PRF (3,000 rpm, 10 min). The clot was stored at −80°C for freezing. The frozen PRF underwent overnight lyophilization at −51°C.	TTCP/OPLC vs. TTCP/OPLC/gelatin vs. TTCP/OPLC/PRF vs. TTCP/OPLC/gelatin/PRF	Incorporated	Mechanical properties: ↑ Ultimate compressive strength ↑ Degradation speed *in vitro* *In vitro*: ↑ Early dental follicle stem cell proliferation ↑ Dental follicle stem cell adhesion ↑ Cell mineralization *in vitro* ↑ Osteogenic differentiation	Anthraper et al. (2024)
	Scaffold - Eggshel HA, collagen, Polylactic Acid–Polyglycolic Acid, PRF	PRF (3,000 rpm, 10 min or 2,700 rpm 12 min)	HAp-egg shell/PLGA, vs. HAp-egg shell/PLGA + collagen vs. HAp-egg shell/PLGA + PRF vs. HAp-egg shell/PLGA + PRF + collagen	Impregnated	*In vitro*: ↑ Human periodontal ligament fibroblasts viability ↑ Human periodontal ligament fibroblasts adhesion	Espitia-Quiroz et al. (2022)
*In vivo*	Sticky bone - Collaginated bone graft with and deprotenized bone graft	PRF (3,000 rpm, 10 min)	Collaginated bone graft with +PRF vs. collaginated bone graft vs. deprotenized bone graft + PRF vs. deprotenized bone graft	Mixing	*In vivo*: ↑ Residual bone graft ↓ New bone formation ↓Osteogenic differentiation *in vivo*	Peker et al. (2016)
	Sticky bone - Mineralized collagen	PRF (1,300 rpm, 14 min)	Material vs. material + PRF	Mixing	*In vivo*: ↑Faster new bone formation ↑ Bone % volume ↓ Residual graft material	Zhang et al. (2023a)
	Sticky bone - Multi-walled carbon nanotube, HA	PRF (400 × g, 12 min)	Control vs. PRF vs. material vs. Material +PRF	Mixing	*In vivo*: = New bone formation ↓ Residual graft material	Bastami et al. (2022)
	Hydrogel - Gelatin, Carbonated HA, A-PRF	A-PRF (1,500 rpm, 14 min). The fibrin clot was then pressed with a PRF processing box for 10 min, as the extracted supernatant was used for material preparation.	Control vs. material vs. material +PRF	Incorporated	*In vivo*: ↑ OPG expression ↑ Tooth position stability after orthodontic appliance removal ↓ RANKL expression	Alhasyimi et al. (2017)
	Hydrogel - Gelatin, Carbonated HA, A-PRF	A-PRF (1,500 rpm, 14 min) The fibrin clot was pressed with a PRF processing box for 10 min, the extracted supernatant was used for material preparation.	Control vs. material vs. material +PRF	Incorporated	*In vivo*: ↑ Osteoblast activity ↓ Osteoclast activity ↓ Tooth relapse after orthodontic appliance removal	Alhasyimi et al. (2018)
	Scaffold - PCL, gelatin, chitosan, poly (γ-glutamic acid), HA	PRF (3,000 rpm, 10 min)	Control vs. chitosan/poly (γ-glutamic acid)/hydroxyapatite vs. chitosan/poly (γ-glutamic acid) vs. chitosan/poly (γ-glutamic acid)/hydroxyapatite + PRF	Incorporated	*In vitro*: ↑ Human dental pulp stem cell viability ↑ Human dental pulp stem cell viability osteogenic differentiation *In vivo*: ↑ Tissue mineralization ↑Osteoblast activity ↑ New alveolar bone formation	Zhang et al. (2019a)
	Scaffold - magnesium phosphate, strontium	Decellularized PRF (3,500 rpm, 15 min). PRF immersed in tris buffer and subjected to 5 freeze-thaw cycles. After, PRF is immersed in 0.25% trypsin/0.01% EDTA, treated with 20 ng/mL Dnase I and 20 ng/mL Rnase A for 16 h, and rinsed with PBS.	MgP vs. MgP + Strontium vs. MgP + Strontium + DPRF	Coated	Mechanical properties: = Ultimate compressive strength *In vivo*: ↑ Percentage of new bone formation ↓ New bone formation time	Tarif et al. (2023)
Studies incorporating PRF into polymer-polymer composites						
*In vitro*	Hydrogel - methacrylated collagen + methacrylated chitosan + PRFe	A-PRF (1,500 rpm, 14 min) Samples homogenized, stored for 20 h at 4°C, stirred at 150 rpm, 37°C, 4 h. The obtained mixture centrifuged at 3500 *g* for 10 min.	ChitMA/ColMA vs. ChitMA/ColMA + PRF	Incorporated	Mechanical properties: ↑ Degradation rate *in vitro* ↓ Young’s modulus ↓ Hydrogel shrinkage ↓ Pore size ↓ Porosity *In vitro*: ↑ SCAP cell migration ↑ SCAP viability ↑ SCAP odontic differentiation	Noohi et al. (2023)
	Core-shell fiber scaffold - PCL, chitosan, L-PRF	L-PRF (2,700 rpm, 12 min)	PCL vs. PCL/CS vs. PCL/CS/PRF	Incorporation in manufacturing	Mechanical properties: ↑ Porosity ↑ Hydrophilicity ↑ Degradation speed Dulbecco’s Phosphate-Buffered Saline ↑ Tensile strength ↑ Elastic modulus ↓ Fiber diameter ↓ Swelling ratio *In vitro*: ↑ MG-63 cell viability ↑ MG-63 osteogenic differentiation ↑ MG-63 cell mineralization	Rastegar et al. (2021)
	Electrospun nanofibers - polyvinyl alcohol, sodium alginate, lyophilized PRF	Lyophilized PRF (400xg, 10 min)	PVA/SA vs. PVA/SA/PRF	Incorporated	Mechanical properties: ↑ Pore diameter *In vitro*: ↑ MEC3T3-E1 cells viability ↑ MEC3T3-E1 osteogenic differentiation	Nie et al. (2020)
	Membrane - Collagen, chitosan, lyophilized A-PRF	Lyophilized A-PRF (1,500 rpm, 14 min)	TCP vs. collagen vs. chitosan/colagen vs. chitosan/collagen/PRF	Incorporatied	Mechanical properties: ↓ Young’s modulus ↓ Degradation rate *In vitro*: ↑ BMSCs viability ↑ BMSCs osteogenic differentiation	Ansarizadeh et al. (2019)
*In vivo*	Scaffold -chitosan, gelatin, L-PRF	L-PRF (400 × g, 10 min)	Chitosan/Gelatin/PRF vs. Chitosan/gelatin vs. control	Incorporation in manufacturing	*Mechanical properties:* ↑ Pore size ↑ Water uptake ↓ Compression modulus *In vitro*: ↑ BMSCs proliferation ↑ BMSCs Adhesion ↑ BMSCs migration ↑ Cell mineralization ↑ Osteogenic differentiation *In vivo*: ↑ Faster new bone formation *in vivo* ↑ Angiogenesis *in vivo*	Chi et al. (2019)

Results from *in vivo* studies suggest similar results. Only two studies showed that the addition of PRF does not improve new bone formation - Peker et al. studied sticky bone made from collaginated bone graft and L-PRF and deproteinized bone graft with PRF for sinus floor augmentation. The authors saw insignificant differences between groups with and without PRF (Peker et al., 2016). Similarly, Bastami et al. (2022) found only slight, insignificant changes in bone defect healing in sheep using sticky bone made with multi-welled carbon nanotubes, HA, and minced PRF clots. In contrast, Alhasyimi et al. (2017); Alhasyimi et al. (2018) demonstrated that gelatin, carbonated HA, and A-PRF injectable hydrogel retain tooth position longer after orthodontic appliance removal by improving osteoblast activity and inhibiting osteoclast activity. Zhang L. et al. (2019); Zhang L. et al. (2023) triple-layer scaffold, consisting of an electrospun PCL/gelatin top layer and a chitosan/poly (y-glutamic acid)/nHA hydrogel bottom layer, showed enhanced healing in rat and New Zealand white rabbit models, with increased new bone tissue formation, higher OPN protein expression, and improved organization and collagen deposition in histological analyses. Additionally, *in vivo* studies observed quicker degradation of graft materials when PRF was used (Zhang Yue et al., 2023; Bastami et al., 2022). This could be due to the cell-mediated degradation of CaP materials and the PRF-induced promotion of cell migration, leading to faster biomaterial phagocytosis (Chi et al., 2019; Tajvar et al., 2023). Despite the lack of detailed information on the mechanical properties of these scaffolds, the overall results suggest that PRF serves as a beneficial growth factor source, enhancing the osteogenic effects of composite scaffolds.

### 4.2 PRF with polymer composite materials

For polymer-polymer composites, PRF has been incorporated during the fabrication process, similar to the methods shown in Figure 1. Analysis of the mechanical properties of these composites (Table 7) reveals varied effects. Out of five studies on different polymer-based compositions, three reported that adding PRF to biomaterials reduced mechanical durability (Chi et al., 2019; Noohi et al., 2023; Ansarizadeh et al., 2019). However, a PCL/chitosan core-shell fiber scaffold loaded with L-PRF showed increased tensile strength and elastic modulus (Rastegar et al., 2021). Incorporating PRF during scaffold fabrication often results in significant morphological changes. For example, adding decellularized L-PRF into gelatin and chitosan scaffolds, as well as L-PRF into electrospun nanofibers made from polyvinyl alcohol and sodium alginate, increased pore size (Chi et al., 2019; Nie et al., 2020), but L-PRF incorporation into PCL/chitosan core-shell fibers increased scaffold porosity (Rastegar et al., 2021). In contrast, adding PRF extract to methacrylated collagen (ColMa) and methacrylated gelatin (GelMa) hydrogel reduced both pore size and porosity (Noohi et al., 2023). Chi et al. (2019) observed that PRF improved the chitosan/gelatin scaffold water absorption, Noohi et al. (2023) found that PRF extracts reduced ColMa/GelMa hydrogel shrinkage, but Rastegar et al. (2021) noted that L-PRF decreased scaffold swelling ratio.

*In vitro* testing of all the polymer composites with PRF demonstrated positive results, boosted bone mesenchymal stem cell (BMSCs) proliferation, adhesion, and osteogenic differentiation were shown with chitosan/gelatin/L-PRF scaffolds and collagen/chitosan/lyophilized A-PRF membranes (Chi et al., 2019; Ansarizadeh et al., 2019). ColMa/GelMa/PRF enhanced odontic differentiation in stem cells from apical papilla (SCAP) (Noohi et al., 2023). Electrospun nanofibers from polyvinyl alcohol/sodium alginate and incorporated lyophilized L-PRF demonstrated better preosteoblast proliferation and osteogenic differentiation compared to nanofibers without L-PRF (Nie et al., 2020).

Although these biomaterials showed positive *in vitro* results, they lack extensive *in vivo* testing. Only Chi et al. (2019) tested their material in rat models, where micro-CT analysis showed greater bone volume and formation at 4- and 8 weeks post-implantation. Histological evaluation revealed vascularized bone tissue in groups treated with the chitosan/gelatin/L-PRF scaffold after 8 weeks.

These results show that even though PRF significantly improves biomaterial biocompatibility improving osteogenetic and angiogenetic properties, PRF impairs the mechanical properties of the materials.

### 4.3 3D printed PRF composite materials

3D bioprinting involves layer-by-layer deposition of biological materials to create structures mimicking living tissues or organs. This combines 3D printing with regenerative medicine to construct functional biological structures for medical applications (Mamo et al., 2023) Recently, 3D printing has been applied in bone tissue engineering and dentistry, allowing for precise fabrication of structures with biocompatible materials (Hadad et al., 2023; Haleem et al., 2020) It offers solutions for creating custom-designed scaffolds that mimic natural bone architecture and can improve the mechanical and biological properties of composite biomaterials (Tavoni et al., 2021).

Since 2018, several research groups have integrated PRF into bio-inks for 3D printing, demonstrating promising outcomes for both soft and hard tissue regeneration in animal studies (Table 8) (Sui et al., 2023; Song et al., 2018; Yi et al., 2022; Grandjean et al., 2024). Song et al. and Sui et al. have prepared 3D-printed scaffolds with PRF for mimicking bone tissue (Sui et al., 2023; Song et al., 2018). An alginate-gelatin and I-PRF scaffold designed by Yi et al. (2022) was aimed to help heal oral soft tissue. The enhanced biological properties of these scaffolds likely stem from PRF’s growth factors, which enhance cell adhesion through improved surface hydrophilicity (Bjelić and Finšgar, 2021). Second, Song et al. and Yi et al. noted that the printed scaffolds had rougher surfaces which could be due to the incorporated fibrin (Khampieng et al., 2018). However, incorporating PRF into 3D inks presents challenges. Yi et al. (2022) found that adding fresh I-PRF to alginate/gelatin ink decreased viscosity and reduced scaffold compressive strength, whereas Sui et al. (2023) using lyophilized L-PRF in L-PRF/chitosan/hydroxyapatite bio-ink observed increased viscosity. The viscosity of the bioink is not influenced only by the PRF but also the rest of the components. Crosslinking between PRF and the materials can start even before the printing process, requiring careful optimization through physical-chemical experiments before printing (Yi et al., 2022; Grandjean et al., 2024). The addition of lyophilized L-PRF did not improve the scaffold’s mechanical properties and with the increase of L-PRF concentration in the scaffold compressive modulus reduced (Sui et al., 2023). Song et al. (2018) also observed a similar reduction when incorporating PRF granules into BCP/PVA bio-ink. This means that the improvement of biological properties of 3D printed scaffolds by PRF comes with a cost of the materials’ mechanical properties, which limits the potential uses of the material.

**TABLE 8:** Summarized information about studies incorporating PRF into bioinks for 3D printing.

	Bioink composition	PRF preparation protocol	Groups	Type of incorporation	The effect of adding PRF to 3D ink	Ref.
*In vitro*	Chitosan, HA, L-PRF powder	L-PRF (400 × g, 10 min). Fresh PRF was frozen in −80°C overnight and lyophilized for 24 h	CH/HA vs. 0.5% PRF/CH/HA vs. 1% PRF/CH/HA vs. 2.5% PRF/CH/HA	Incorporated	Mechanical properties: ↑ Viscosity of bio-ink = Morphology ↓ Compression modulus ↑ Faster scaffold degradation time = Hydrophilicity = Porosity ↓Compression modulus *In vitro*: ↑ MC3T3-E1 cell proliferation	Sui et al. (2023)
	Sodium alginate, methyl-cellulose, I-PRF	I-PRF (700 rpm, 3 min)	Sodium alginate/methylcellulose/I-PRF vs. Sodium alginate/methyl-cellulose/phosphate buffer saline	Incorporated	*In vitro*: ↑ L929 and SaOS-2 cell viability ↑ Blood vessels growing in length and thickness	Grandjean et al. (2024)
*In vivo*	BCP/PVA/PRF	L-PRF (400× g, 10 min)	Printed BCP/PVA/PRF vs. non-printed BCP/PVA/PRF vs. printed BCP/PVA vs. non-printed BCP/PVA	Incorporated	Mechanical properties: ↑ Surface roughness of the scaffold ↑ Hydrophilicity ↓ Compressive modulus *In vitro*: = BMSCs viability ↑ BMSCs seeding density ↑BMSC adhesion ↑ BMSCs proliferation ↑ BMSCs osteogenic differentiation *In vivo*: ↑ Faster new bone formation *in vivo* ↑ Faster scaffold degradation time *in vivo* ↑ Callus formation	Song et al. (2018)
	I-PRF, alginate, gelatin	I-PRF (700 rpm, 3 min)	Alginate/gelatin vs. Alginate/gelatin/10% PRF vs. Alginate/gelatin/30% PRF vs. Alginate/gelatin/50% PRF	Incorporated	Mechanical properties: ↑ Sol-gel critical temperature ↑ Surface roughness of the scaffold = Degradation *in vitro* ↓ Bioink viscosity ↓ Compression modulus *In vitro*: ↑ Growth factors release time = Human gingival fibroblast viability ↑ Human gingival fibroblast proliferation ↑ ECM production *In vivo*: ↑ Angiogenesis ↑ Host tissue infiltration into scaffolds	Yi et al. (2022)

## 5 Discussion

PRF enhances tissue regeneration across various biomaterials by promoting immunomodulatory protein corona formation, thereby facilitating cell attachment (Fernandez-Medina et al., 2023). The composition of protein corona on a material can impact the cell morphology and viability as well as the release profile of cytokines from the attached cells (Serpooshan et al., 2015). Studies demonstrate that PRF significantly improves cell adhesion (up to 13%) and proliferation, particularly on synthetic polymers (Chi et al., 2019; Al-Maawi et al., 2021; Hoda et al., 2021). Improved osteoblastic differentiation by higher ALP levels is observed in listed types of materials, like zinc, tricalcium phosphate, and xenogenic bone substitute materials (Blatt et al., 2021a; Molenda and Kolmas, 2011). Changes in osteoconduction are also observed as PRF composites improved Collagen I alpha-1 gene expression and calcium mineralization (Zhang L. et al., 2019; Song et al., 2018; Chi et al., 2019; van Orten et al., 2022; Blatt et al., 2021b; Rastegar et al., 2021; Zhang L. et al., 2023). Combining PRF with biomaterials prolongs cytokine release, supporting prolonged cellular activities crucial for tissue regeneration, including proliferation, migration, and differentiation (Sui et al., 2023; Song et al., 2018; Chi et al., 2019; Blatt et al., 2021a; Zheng et al., 2015; Le et al., 2023). This sustained release promotes persistent angiogenic responses, crucial for supporting blood vessel formation in damaged tissues and enhancing overall tissue regeneration success (Nurkesh et al., 2020; Ucuzian et al., 2010). The higher concentrations and prolonged release of growth factors from PRF compared to whole blood likely contribute to pronounced blood vessel formation and increased branching points when PRF is integrated with materials (Sebastian et al., 2022; Yi et al., 2022; Blatt et al., 2020; Nishimoto et al., 2015; Egle et al., 2021).

When looking at these composites in a bigger picture – *in vivo* and clinical studies show mostly positive results. In clinical studies, the most tested materials are CaP, bioglass, silk, collagen, and xenogenic bone grafts. Research of composite materials with PRF is limited to animal *in vivo* studies. The most coherent observations from these studies are that the PRF reduces the necessary time for new bone tissue formation (Zhang L. et al., 2019; Yilmaz et al., 2014; Tiwari et al., 2020; Zhang L. et al., 2023; Baghele et al., 2023; Abd-Elkawi et al., 2023). This acceleration is particularly evident during early healing stages, and control groups without PRF tend to achieve similar tissue formation levels in later stages (Song et al., 2018; Tarif et al., 2023; Abdullah, 2016; Alkafarani and Baban, 2019; Zhang Yue et al., 2023). Similarly, the addition of PRF reduces healing time after dental implant insertion and improves its stability (Hwan Jung et al., 2020; Pichotano et al., 2018; Pichotano et al., 2019; Ramy Salah et al., 2021; Angelo et al., 2015; Tabrizi et al., 2018; Potres et al., 2016; Mohamed Abdel-Aziz et al., 2023). These findings are crucial as they potentially alleviate healthcare burdens by minimizing patient recovery periods and reducing the duration of healthcare facility stays (Sen, 2021).

*In vivo* experiments have shown that biomaterials degrade more rapidly when combined with PRF, particularly noted in studies involving CaPs and their composites (Song et al., 2018; Nizam et al., 2018; Zhang Yue et al., 2023; Bastami et al., 2022). The reason for the observed effect of PRF on biomaterial degradation is still unknown and worth studying in future research. Several mechanisms may contribute to this phenomenon, including hydrolytic deposition, cell-mediated degradation, and loss of scaffold integrity due to mechanical stresses (Tajvar et al., 2023). One plausible mechanism involves leukocytes present in PRF, which can generate reactive oxygen species (ROS) such as hydrogen peroxide (H_2_O_2_), nitric oxide (NO), and superoxide (O_2−_). These ROS can degrade biomaterials by initiating hydrogen atom separation from polymer chains and initiating propagation reactions (Tajvar et al., 2023). Additionally, PRF contains matrix metalloproteinases (MMPs) that are involved in tissue remodeling and can contribute to collagen and its derivative degradation (Tajvar et al., 2023; Eren et al., 2016; Stamenkovic, 2003). In bioceramic degradation, a big role is played by osteoclasts that absorb CaPs like bone minerals (Tajvar et al., 2023). Unfortunately, PRF effects on osteoclastogenesis are inconclusive. Multiple studies show that PRF inhibits osteoclast activity and differentiation (Kumar et al., 2021; Kumar et al., 2019; Kargarpour et al., 2020), while others show that PRF mixing with biomaterials induces higher levels of proinflammatory cytokines (IL-6 and TNF-α) that can activate osteoclastogenesis (Park et al., 2023).

While many studies report positive outcomes from incorporating PRF into biomaterials, there remain inconclusive results, possibly due to variations in PRF protocol types. This article identifies specific PRF protocols used in included studies to explore how these choices affect tissue regeneration, though not all publications provided detailed PRF protocols. Adding to this issue, authors frequently deviate from established protocols. Commonly used PRF protocols include L-PRF (3,000 rpm or 400 g for 10 min), A-PRF (1,500 rpm for 14 min), and I-PRF (700 rpm for 3 min) (Dohan et al., 2006; Miron et al., 2017; Ghanaati et al., 2014). However, variations such as using 2,700 rpm for 3 min for I-PRF or 3,500 rpm for 15 min for clot-type PRF have been observed, complicating result analysis (Tarif et al., 2023; abou shabana et al., 2023; Fabbro et al., 2013). High centrifugation speeds (higher than 400 × g) for longer than 8 min reduce the leukocyte and platelet concentration for clot-type PRFs which could alter the healing properties (Miron et al., 2020). Moreover, differences in centrifuge equipment, including vibration frequencies, can impact cell populations within these clots (Dohan et al., 2018). Additionally, variations in platelet counts can occur both between individuals and within the same individual at different times of the day (Mazzocca et al., 2012). In conclusion, while combining PRF with bone graft materials shows promise for enhancing bone regeneration and healing, findings vary among studies. Standardized protocols and more extensive clinical trials are essential to fully understand and optimize these combinations. Researchers and clinicians should consider the concept of lower centrifugation speeds to maximize growth factor concentrations. The method of incorporating PRF into materials is also crucial; distributing PRF throughout the material prolongs its bioactive effects due to physical constraints imposed by scaffolds. Integration of PRF with scaffolds influences their physicochemical properties, necessitating thorough experimental studies to determine suitable mechanical and biological properties for specific procedures.
